# Sinapic acid attenuated nephrotoxicity against Cyclophosphamide in mice model: A histochemical, immunohistochemical and histopathological evaluation 

**DOI:** 10.22038/ijbms.2025.83903.18155

**Published:** 2025

**Authors:** Saeed Raoof, Shiva Rezaei, Mehryar Zargari, Mansoureh Mirzaei, Seyed Jalal Hosseinimehr, Abbasali Karimpour Malekshah, Fereshteh Talebpour Amiri

**Affiliations:** 1 Department of Anatomy, Faculty of Medicine, Molecular and Cell Biology Research Center, Mazandaran University of Medical Sciences, Sari, Iran; 2 Student Research Committee, Faculty of Medicine, Mazandaran University of Medical Sciences, Sari, Iran; 3 Department of Biochemistry, Faculty of Medicine, Mazandaran University of Medical Sciences, Sari, Iran; 4 Department of Radiopharmacy, Faculty of Pharmacy, Mazandaran University of Medical Sciences, Sari, Iran

**Keywords:** Caspase-3, Cyclophosphamide, Nephrotoxicity, NF- kB, Oxidative stress, Sinapic acid

## Abstract

**Objective(s):**

Cyclophosphamide (CP) is a chemotherapeutic drug used to treat various tumors. It causes nephrotoxicity by producing reactive oxygen species. Sinapic acid (SA) exhibits anti-oxidant, antiapoptotic, and anti-inflammatory activities at low doses as a phenylpropanoid. This study aimed to investigate the protective effects of SA on SP-induced renal injury.

**Materials and Methods:**

Forty-eight BALB/c mice were randomly divided into control, SA (for seven consecutive days, with two doses of 5 and 10 mg/kg), CP (single dose, 200 mg/kg), and CP + SA (5 and 10 mg/kg). On the 10th day of the study, mice were examined by renal function markers (Urea and Creatinine), oxidative stress markers (MDA and GSH), histopathological, and immunohistochemical assays (caspase-3 and NF-kB kidney).

**Results:**

MDA levels increased and GSH levels decreased significantly in CP-treated mice. In addition, the histopathological structure of the kidney tissue in CP-treated mice showed significantly severe kidney tissue damage associated with increased urea and creatinine. The administration of SA in CP-treated mice significantly reduced serum urea and creatinine concentrations. In addition, the immunohistochemical staining of caspase- 3 and NF-kB decreased significantly in the CP + SA group compared to CP-treated mice.

**Conclusion:**

Overall, our study suggests that sinapic acid, a substance with antioxidant, antiapoptotic, and anti-inflammatory properties, can be used as a complementary therapy to protect nephrotoxicity against CP.

## Introduction

Alkylating agents, some of the oldest anticancer compounds with a DNA damage mechanism, are still widely used (1). Cyclophosphamide (CP) is an alkylating agent famous for treating cancers, sarcomas, hematological malignancies, and autoimmune diseases (2). Nephrotoxicity is created by CP attributed to its toxic metabolite, acrolein, produced during CP activation (3). It has been proven that CP-induced kidney injury by producing reactive oxygen species (ROS) and nitric oxide (NO) eventually causes lipid peroxidation, protein oxidation, and DNA damage (3). The body’s physiological activities continuously produce ROS. Oxidative stress is the condition in which the excess manufacture of ROS overcomes the anti-oxidant defense (4). The kidney is one of the important organs in the metabolism and excretion of CP and its metabolites; therefore, damage to this organ is considered a side effect of CP, which has limited its use (5). According to previous studies, oxidative stress caused a significant decrease in Glutathione (GSH) content and a significant increase in Malondialdehyde (MDA) levels. GSH, the most prominent anti-oxidant compound in cells and body fluids, and MDA, the final lipid peroxidation product, are considered oxidative stress markers (6). CP also increased serum levels of urea and creatinine. Creatinine level is a measure of glomerular filtration function and kidney function. It was shown that CP prevents the purification of creatinine from the blood by disrupting the function of the glomerular system. During the metabolism of proteins, ammonia in the liver causes the production of urea, which is excreted through the kidneys. Other harmful effects of CP include excessive retention of urea in the blood and disruption of the ratio of urea production to its clearance (7). Past histopathological investigations demonstrated that CP causes pathological changes in kidney tissue, including renal tubular dilatation, infiltration of acute inflammatory cells, interstitial edema, coagulative necrosis, and dilation and congestion of renal blood vessels. Among other harmful effects of CP, we can mention the increase in the activity of apoptosis and inflammatory markers (8).

Polyphenols are compounds found in plants that have been studied for their antioxidant properties and health benefits (9). Sinapic acid (SA) is one of the hydroxycinnamic acids commonly found in many plant foods, such as vegetables, grains, fruits, spices, oilseeds, and berries. SA has also shown anti-oxidant, anti-inflammatory, anti-cancer, anti-apoptotic, anxiolytic, and anti-hyperglycemic effects (10). In addition, SA, as a phytochemical, has been reported to be effective against benzo(A) pyrene-induced lung cancer (11), methotrexate-induced liver injury (12), cisplatin-induced nephrotoxicity (13). 

In this study, we determined the effects of SA on nephrotoxicity against CP. For this purpose, we examined tissue (oxidative stress markers), blood (urea and creatinine), and histotological examinations of mice.

## Materials and Methods

### Chemicals

CP was purchased from Baxter (Germany) and SA from Sigma-Aldrich (St. Louis, MO, USA). SA was readied with 5% carboxymethyl cellulose and then used 0.5 cc per animal.

### Animals

In this study, the number of mice was forty-eight, with each weighing between 25 and 30 grams. All subjects were male BALB/c mice. The mice were from the Animal Research Center of Mazandaran University of Medical Sciences and retained under standard conditions (temperature 23 ± 2 °C, moisture 55 ± 5%, 12 hr light/dark phase). The animal’s access to food and water was according to the protocol of the animal care center. All steps concerning this study were acted following the directions of the Animal Ethics Committee.

### Experimental design

The mice were randomly divided into six groups:

Control group: The control group received oral equivalent saline for seven consecutive days.

SA5 and SA10: Sinapic acid was given orally in two doses of 5 and 10 mg/kg for seven successive days following the start of the study.

CP group: CP 200 mg/kg was injected intraperitoneally as a single dose on the study’s third day.

SA 5 + CP and SA 10 + CP group: On the third day, animals were injected intraperitoneally with CP 200 mg/kg. They were then simultaneously treated with SA 5 and 10 mg/kg orally for 7 days **(**14**).**

**Figure 1:**
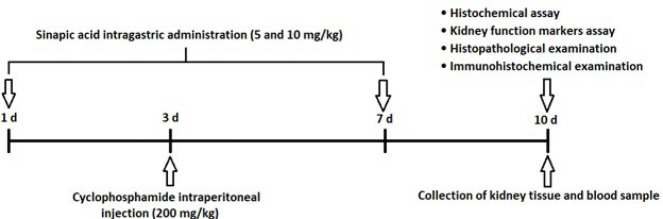
Experimental design diagram of the effect of sinapic acid on cyclophosphamide-induced kidney injury in mice

The experimental diagram and the steps of the steps are proved in Figure 1.

### Sampling and biochemistry

In the tenth day of the study, the animals were anesthetized with ketamine and xylazine. The blood samples from the heart were centrifuged for 15 min at 3000 rpm to separate the serum. Then, serum samples for urea and creatinine measurements were stored at −80 °C. After exiting the kidney, remove excess tissue and wash the kidney with phosphate-buffered saline (PBS). The right kidney was frozen with liquid nitrogen and immediately stored at −80 °C for MDA and GSH analysis. The left kidney was fixed in 10% buffered formalin to prepare histological and immunohistochemical sections.

### Tissue biochemical assay

Thiobarbituric acid (TBA) was used to determine MDA levels as the end product of lipid peroxidation in kidney tissue. First, the sample was homogenized. Then, TBA, phosphoric acid, and water purified by distillation were added to the sample and boiled for 45 min. After the temperature had dropped, n-butanol was added to the sample to extract the TBA cooling reactant. In the next step, the n-butanol layer was separated by centrifugation (3500 rpm, 10 min), and its density was determined spectrophotometrically. After drawing the MDA standard curve, the MDA level was obtained in nmol/mg tissue. The GSH content in the sample was calculated using 5,5’-dithiobis-2-nitrobenzoic acid (DTNB) as an index. 

The GSH concentration was calculated spectrophotometrically (412 nm) as μmol/g tissue (14).

### Assay of urea and creatinine

The amount of urea and creatinine in the blood as markers that show the level of kidney health was evaluated using a biochemical spectrophotometry kit (Pars Azmon, Iran).

### Histological examination

Mice kidneys were fixed in 10% formalin buffer for 24 hr, then dehydrated, cleared in xylene, and embedded in paraffin. Then, 5 μm thick sections were prepared using a microtome. The sections were then stained with hematoxylin and eosin (H&E), and a histologist performed a blinded histopathological examination under a microscope (Olympus, Japan). The kidney injury was used as a partial assessment to determine the degree of kidney injury. Five images were taken from each segment in each group to examine renal tubular dilatation, Bowman space, epithelial cell apoptosis, and necrosis. The score according to the percentage of tubular damage is 0: none, 1: <25%, 2: 25–50%, 3: 50–75%, 4: > 75% (14).

### Immunohistochemical examination

Immunohistochemical detection of caspase-3 and NF-κB was performed according to the company’s instructions (Abcam, USA). First, the sections were deparaffinized with xylene, rehydrated using an alcohol series, and endogenous peroxidase activity was blocked in 5 μm thick sections with a 0.3% H_2_O_2_ methanol solution. The sections were then incubated overnight with a primary antibody (anti-rabbit polyclonal, 1:75, v/v, Abcam, lot: GR224831-2 and 831054 ab7970-1).The sections were then incubated with a secondary antibody conjugated to horseradish peroxidase (mouse and rabbit-specific HRP/DAB, Abcam, Lat GR2623314-4) for 2 hr. After 5 min incubation with diaminobenzidine tetrahydrochloride, slides were dehydrated and mounted. Finally, quantitative analysis and visual evaluation of immunohistochemistry micrographs were performed using Mac Biophotonics ImageJ 1.41a software (15).

### Statistical analysis

Data were analyzed, and group comparisons were performed using a one-way ANOVA analysis of variance and Tukey’s *post hoc* test on GraphPad Prism software (version 9). Data are expressed as mean ± standard deviation. *P*<0.05 was considered significant.

## Results

### Effects of sinapic acid on MDA and GSH

**Figure 2:**
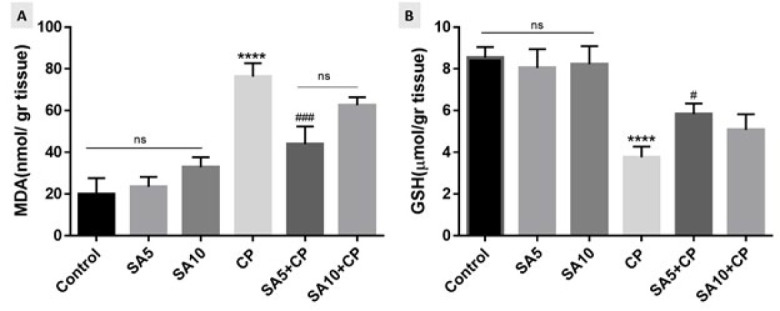
Effect of SA on MDA (Figure A) and GSH (Figure B) levels in mice kidney

The levels of MDA and GSH in renal mice are shown in Figure 2. As an index of lipid peroxidation, MDA was significantly elevated in the CP-treated group compared with the control group (*P*<0.05). In addition, SA administration in CP-treated mice reduced the MDA levels in the tissue. The results showed that the effect of SA was dose-dependent, and SA at a dose of 5 mg/kg was the most effective in reducing MDA levels in the CP-treated group (Figure 2A). GSH levels in mice receiving CP were significantly decreased compared to the control group (*P*<0.05). On the other hand, mice treated with SA and CP compared to the alone CP group had a significant increase in GSH level, which indicates the effect of SA on improving the anti-oxidant defense in the presence of CP as an inducer of oxidative stress. Furthermore, the administration of SA at a dose of 5 mg/kg compared to SA at a dose of 10 mg/kg had GSH levels closer to the control group (Figure 2B).

### Effects of sinapic acid on kidney function markers

Blood urea and creatinine levels as indicators of renal damage in each group are shown in Table 1. The data showed a significant increase in urea and creatinine in the CP-treated mice compared to the control group (*P*˂0.0001). It was also seen that blood urea and creatinine levels decreased significantly in the CP-treated mice receiving SA (*P*˂0.05). Among the two SA doses, 5 mg/kg SA could produce lower urea and creatinine levels and was more effective in reducing renal damage.

### Histopathological results

**Figure 3:**
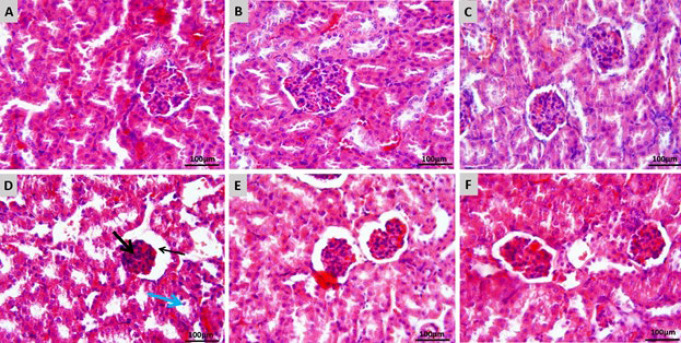
Effects of SA on CP-induced kidney injuries in mice

Figure 3 shows the histological patterns of kidney tissues in each group. When the kidney tissue was examined in the control group, glomerular and tubular histological structures were found to be normal (Figure 3A). In addition, normal kidney tissue structure was observed in mice receiving 5 and 10 mg/kg SA (Figure 3B and C). Histological evaluation showed that mice treated with CP showed dilatation in the renal tubular lumen and Bowman’s capsule. The glomerular network was severely atrophied, desquamation of tubular epithelial cells was seen, and congestion of the glomerular vascular network and interstitial tissue was evident, indicating severe renal tissue damage (Figure 3D). These changes were more limited in the group receiving SA 5 mg/kg + CP, with mild tubular dilatation, mild dilatation of Bowman’s capsule, and mild glomerular atrophy (Figure 3E). In addition, moderate dilatation of renal tubules and Bowman’s capsule and moderate glomerular atrophy were observed in CP-treated mice receiving SA 10 mg/kg + CP (Figure 3F). In general, it was found that the kidney tissue of receiving CP mice given 5 and 10 mg/kg SA was better, but treatment with the SA 5 mg/kg dose resulted in greater recovery than the SA 10 mg/kg dose.

**Figure 4:**
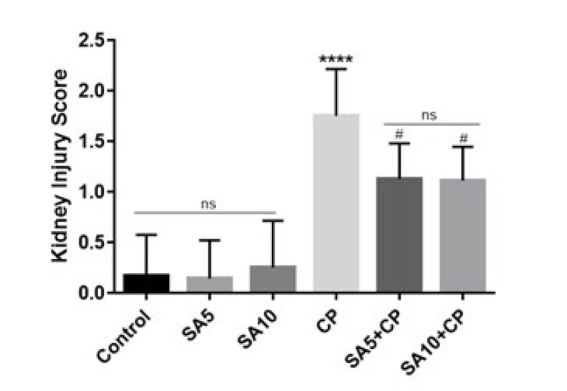
Kidney injury score

The kidney injury score is shown in Figure 4. The results showed that CP significantly increased the kidney injury score compared to the control group (*P*˂0.0001). Treatment with CP and SA at doses of 5 and 10 mg/kg also significantly reduced the kidney injury score compared with the group receiving CP alone (*P*˂0.05).

### Immunohistochemical findings

**Figure 5:**
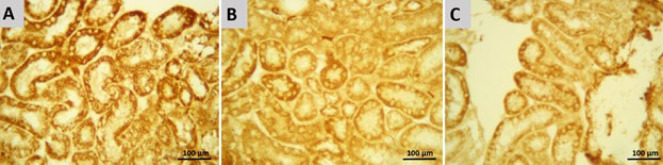
Immunohistochemical staining of caspase-3 in Renal epithelial cells of mice

**Figure 6:**
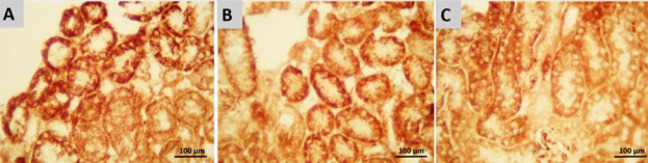
Immunohistochemical staining of NF-kB in renal epithelial cells of mice

Immunohistochemical analysis of the kidney used caspase-3 as a marker of apoptosis (Figure 5). The staining intensity of positive cells (brown color) indicates caspase-3 immunoreactivity. In CP-treated mice, positive cells, including epithelial cells of cortical and medullary tubules, were found (Figure 5A), and caspase-3 immunoreactivity was significantly increased compared with the control (*P*<0.0001). On the other hand, SA treatment with 5 and 10 mg/kg + CP significantly reduced caspase-3 immunoreactivity of mice renal epithelial cells compared with the CP-treated group (*P*<0.05 and *P*<0.01, respectively). According to observations, it was found that 5 dose of SA played a better role in reducing caspase-3 immunoreactivity and reducing overall renal tissue apoptosis than 10 dose of SA (Figure 5B and 5C). Another immunohistochemical stain indicated inflammation by measuring NF-κB (Figure 6). Cortical and medullary tubular epithelial cells were significantly stained in CP-treated mice (Figure 6A), indicating that NF-κB expression was increased in them compared with the control group (*P*<0.05). NF-κB immunoreactivity was significantly diminished in the SA 5 and 10 mg/kg group compared with the CP-treated group (*P*<0.0001). Among the two SA doses, the 5 mg/kg dose was more effective than the 10 mg/kg dose in reducing NF-κB immunoreactivity (*P*<0.001 and *P*<0.0001, respectively) (Figures 6B and 6C).

### Semiquantitative analysis of immunohistochemical staining

**Figure 7:**
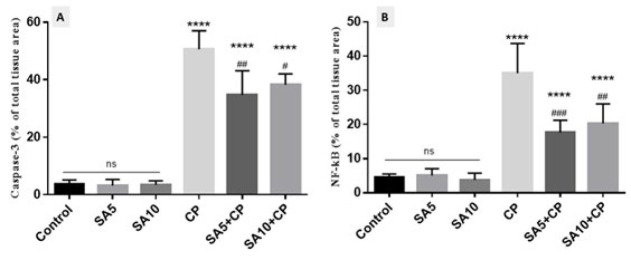
Histograms of the semiquantitative analysis of immunohistochemical staining for Caspase-3 and NF-kB

Semiquantitative analysis and densitometric evaluation of caspase-3 and NF-κB staining in all groups are shown by the histogram in Figure 7. According to the semiquantitative analysis, the group of CP-treated mice evidences the most intense immunoreactivity of caspase-3 and NF-kB (50.6±6.4 and 35±8.7, respectively) compared to other groups (*P*<0.0001). SA administration in CP-induced mice resulted in lower caspase-3 (34.7±8.4 and 38.2±3.8, respectively) and NF-kB immunoreactivity (17.6.7±3.6 and 20.3±5.7, respectively) levels compared with mice receiving CP only (*P*<0.05). The expression levels of caspase-3 and NF-kB were the same in the control and SA groups.

## Discussion

Numerous studies have shown that CP, a chemotherapy drug, can harm healthy tissues. To explore new treatment options for cancer patients, this study investigated the antioxidant and anti-apoptotic effects of SA in a mouse model of nephrotoxicity induced by CP. The findings indicate that SA exhibits antioxidant, anti-apoptotic, and anti-inflammatory properties, which reduce oxidative stress, improve histopathological features, reduce urea and creatinine levels, and enhance immunoreactivity of Caspase-3 and NF-kB. 

The use of beneficial compounds as a complementary therapy is one of the ways that can be adopted to reduce the nephrotoxicity caused by CP. Previous studies have confirmed the effect of SA on nephrotoxicity induced by drugs such as Cisplatin (16), Cadmium (17), Gentamicin (18), Lead acetate (19) and 5-fluorouracil (20) resistance.

SA has been proven to be a good free radical scavenger. SA exhibits antioxidant, anti-inflammatory, and antiapoptotic effects through various mechanisms, such as scavenging of free radicals or protecting intracellular antioxidant enzymes (20). During oxidative stress, ROS initiates the cycle of lipid peroxidation, during which peroxyl radicals are created. Peroxyl radicals cause damage to enzymes and membrane proteins and begin the cycle of lipid peroxidation again (21). MDA is one of the eventual products of lipid peroxidation, which leads to the destruction of membrane lipids by oxygen uptake enhancement, increasing membrane permeability to Ca+2 ions and lipid radicals’ formation (22). In our examination like another study, it has been observed CP administration increases the content of MDA. Also, the level of glutathione (GSH), which is responsible for the detoxification of endogenous and exogenous toxic substances, was decreased in the CP group (21). According to a previous study, SA administration in CP-treated mice mitigated MDA and increased GSH in the kidney tissue (13). In line with our study, Ansari et al used SA 10 and 20 mg/kg orally for 2 weeks to reduce the nephrotoxicity induced by cisplatin, and the higher dose of SA (20 mg/kg) was more effective (13). While in our pilot study, we have seen that a dose of 20 mg/kg has a slight deleterious effect in histopathological evaluation. Therefore, we used two doses of 5 and 10 mg/kg and in our study, the best dose was the lowest dose of SA (5 mg/kg).

Urea and creatinine are two biochemical enzymes in the blood that are widely known as kidney function markers. Oxidative stress induced by CP, due to kidney damage and increased membrane permeability, causes urea and creatinine to leak into the blood circulation and augmentation the serum level of these two (23). The present analysis showed that urea and creatinine levels were significantly increased in CP-treated mice. like our result, it has been confirmed that treatment with SA prevented kidney damage by reducing urea and creatinine (24).

To confirm that the kidney was damaged by CP, we examined the changes in kidney tissue, which showed dilatation of renal tubules and Bowman’s capsule, severe glomerular atrophy, epithelial shedding, and vascular occlusion in the glomerular and interstitial tissues. According to previous histopathological observations, SA exerts a protective effect by suppressing these changes (25). In apoptosis, cells die in a programmed manner to maintain tissue homeostasis and demonstrated using apoptotic (such as caspase-3 and Bax) and antiapoptotic (such as Bcl-2) markers (26). In a previous immunohistochemical analysis, similar to our results, caspase-3 was activated in CP-treated mice and caused apoptosis in kidney tissue (27). Here, SA treatment prevented apoptosis in the CP-treated group, suggesting that SA has anti-apoptotic properties (28). Alaofi et al have shown that SA treatment may exerts its anti-apoptotic effecs by decreasing other apoptotic markers such as Bax and increasing anti-apoptotic markers such as Bcl-2 (24). In oxidative stress, ROS stimulate NF-kB, which itself regulates other proinflammatory factors such as interleukin-6, interleukin-1β, and tumor necrosis factor-α (TNF-α) (29). Consistent with our findings, previous studies have shown that CP stain more for NF-κB, which is indicative of kidney disease (30). In our data, as in other studies, SA treatment reduced the immunological expression of NF-κB, a marker of inflammation (18).

**Table 1:** Effect of SA, CP, and CP+SA on urea and creatinine levels

Groups	Urea(mg/dl)	Creatine(mg/dl)
Control	43/7 ± 7/6	0/4 ± 0/1
SA(5 mg/kg)	44/3 ± 5/7	0/4 ± 0/06
SA(10 mg/kg)	42/3 ± 8/1	0/4 ± 0/09
CP	70/7 ± 5**	0/7 ± 0/06***
CP+ SA(5 mg/kg)	52 ± 1/7^#^	0/6 ± 0/06^#^
CP+ SA(10 mg/kg)	55/3 ± 5/9	0/5 ± 0/06^#^

All values are expressed as mean ± SD. * significant vs control; # significant vs CP group. CP: Cyclophosphamide; SA: Sinapic acid. #*P*<0.05; ##*P*<0.01; ***P<0.001.

Data are expressed as mean ± SD. *significant difference versus control group; #significant difference versus CP group. MDA: Malondialdehyde; SA: Sinapic acid; CP,: Cyclophosphamide; GSH: Glutathione, PIP: Piperine.. * significant vs control; # significant vs CP group. #*P*<0.05; ###*P*<0.001; ****P<0.0001.

(A) Control group; kidney tissue in normal histological structure. (B) SA 5 mg/kg and (C) SA 10 mg/kg groups; kidney tissue in normal histological structure. (D) CP group; dilatation in the renal tubule and bowman capsule (thin black arrow), Severe glomerular atrophy (thick black arrow), epithelial desquamation (blue arrow), and hyperemia in glomerulus and interstitial tissue vessels. (E) SA 5 mg/kg + CP group; slight dilatation in the renal tubule, mild dilatation in the bowman capsule, and mild glomerular atrophy. (F) SA 10 mg/kg + CP Group; no significant change in the histological architecture of the kidney. H&E staining; 20× magnification; Scale bar = 100 μm. CP: Cyclophosphamide; SA: Sinapic acid

The scoring based on the percentage of kidney tubular damage: 0: none; 1: <25%; 2: 25–50%; 3: 50–75%; 4: >75%. Data are expressed as mean ± SD. *significant difference versus control group; #significant difference versus CP group. SA: Sinapic acid; CP: Cyclophosphamide. #*P*<0.05; ****P<0.0001.

(A) CP group; Increase in caspase-3 immunoreactivities significantly compared to the control group. (B) SA 5 mg/kg + CP group. (C) SA 10 mg/kg + CP Group; significant reduction in immunoreactivity of caspase-3 compared to CP-treated group. The brown color shows caspase-3 positive cells. 20× magnification; Scale bar = 100 μm. CP: Cyclophosphamide; SA: Sinapic acid

(A) CP group; Increase in NF-kB immunoreactivities significantly compared to the control group. (B) SA 5 mg/kg + CP group and (C) SA 10 mg/kg + CP Group; significant reduction in immunoreactivity of NF-kB compared to CP-treated group. The brown color shows NF-kB-positive cells. 20× magnification; Scale bar = 100 μm. CP: Cyclophosphamide; SA: Sinapic acid

Data were presented as a percentage of the total tissue area. *significant difference versus control group; #significant difference versus CP group. SA: Sinapic acid; CP: Cyclophosphamide. #*P*<0.05; ##*P*<0.01; ###*P*<0.001; ****P<0.0001.

## Conclusion

In this study, SA can reduce oxidative stress (reduce MDA and increase of GSH), improve renal function (reduce Urea and Creatinine), protect renal tissue, reduce apoptosis (downgrade of caspase-3 staining), reduce inflammation (reduce NF-kB reactivity), and demonstrate its anti-oxidant, anti-inflammatory and antiapoptotic effects to prevent CP-induced renal injury by CP. In conclusion, we demonstrated for the first time that SA is protective against CP-induced nephrotoxicity. SA inhibits CP-induced inflammation and apoptosis by reducing oxidative stress mechanisms and improving renal tissue structure.

## Declaration

We have not used any AI tools or technologies to prepare this manuscript.
